# Associations between malaria-related ideational factors and care-seeking behavior for fever among children under five in Mali, Nigeria, and Madagascar

**DOI:** 10.1371/journal.pone.0191079

**Published:** 2018-01-25

**Authors:** Mai Do, Stella Babalola, Grace Awantang, Michael Toso, Nan Lewicky, Andrew Tompsett

**Affiliations:** 1 Department of Global Community Health and Behavioral Sciences, Tulane University School of Public Health and Tropical Medicine, New Orleans, Louisiana, United States of America; 2 Johns Hopkins Center for Communication Programs/Department of Health, Behavior and Society, Johns Hopkins University, Baltimore, Maryland, United States of America; 3 USAID/President’s Malaria Initiative, Washington, District of Columbia, United States of America; Centro de Pesquisas Rene Rachou, BRAZIL

## Abstract

Malaria remains one of the leading causes of morbidity and mortality among children under five years old in many low- and middle-income countries. In this study, we examined how malaria-related ideational factors may influence care-seeking behavior among female caregivers of children under five with fever. Data came from population-based surveys conducted in 2014–2015 by U.S. Agency for International Development-funded surveys in Madagascar, Mali, and Nigeria. The outcome of interest was whether a child under five with fever within two weeks prior to the survey was brought to a formal health facility for care. Results show a wide variation in care-seeking practices for children under five with fever across countries. Seeking care for febrile children under five in the formal health sector is far from a norm in the study countries. Important ideational factors associated with care-seeking behavior included caregivers’ perceived social norms regarding treatment of fever among children under five in Nigeria and Madagascar, and caregiver’s knowledge of the cause of malaria in Mali. Findings indicate that messages aimed to increase malaria-related knowledge should be tailored to the specific country, and that interventions designed to influence social norms about care-seeking are likely to result in increased care-seeking behavior for fever in children under five.

## Introduction

Malaria remains one of the major causes of death and illnesses in children under five in many low- and middle-income countries [1, 2], despite the availability of simple inexpensive prevention and treatment [3, 4]. In 2015, sub-Saharan Africa was home to 90% of cases and 92% of deaths from malaria worldwide, of which about 70% were among children under five [1]. In Madagascar, Mali, and Nigeria, malaria continues to be a major cause of child illness and death. For example, in Mali, more than two-thirds of deaths in children under five were attributed to malaria [5]. In 2015, the prevalence of malaria among Malian children under five was 36% based on microscopy and 32% based on rapid diagnostics tests [5]. In Nigeria, a quarter of all infant deaths and almost a third (30%) of deaths in children under five were due to malaria [6]. The burden of malaria continues to have serious health and socioeconomic consequences in the region [7]. The risk of malaria infection varies across geographic malaria transmission areas in Mali and Madagascar, while everyone is considered at risk in Nigeria.

Access and utilization of malaria-related services in the formal sector in all three countries also varies widely. For example, Littrell et al. [8] reported fever treatment with artemisinin-based combination therapies (ACTs) among children under five at only 3% in Madagascar and 5% in Nigeria. In some areas of Mali, children with malaria were generally first treated at home, often with herbal remedies, resulting in complications and high mortality rates [9–11]. In Nigeria, despite long-term commitment from the government and significant support from donors, there remains much room for improving the prevalence of malaria-related behaviors. For example, the most recent Demographic and Health Survey (DHS) reported that while most caregivers sought advice for children with fever, prompt treatment with ACTs was rare [12]. Bedford and Sharkey [13] also reported findings from a qualitative study from two Nigerian states in which primary caregivers of children under five faced physical and economic barriers to accessing to health services as well as socio-cultural barriers, such as gender dynamics and low caregiver knowledge of the causation and prevention of childhood illnesses. The quality of malaria treatment and management practices by health-care providers was generally low [14] and varied greatly between different types of health-care providers [15]. Despite being perceived by caregivers as the most easily accessible providers for malaria treatment, patent medicine vendors (PMVs) have been found to provide the lowest technical quality of care [16–18]. Caregivers in southeast Nigeria were also least satisfied with PMVs and most satisfied with public and private hospital service providers; urban caregivers were generally more satisfied with health services than rural caregivers [15]. Few studies have examined sources of malaria treatment services in Madagascar. The most recent DHS found that caregivers sought care for 41.4% of children with recent fever at a health-care facility or from a health-care provider. This figure excluded fever treatment from pharmacies, drug sellers, and traditional healers [19]. All three countries have much room for improvement in care-seeking for fever among children under five. To that end, this study aims to examine the ideational factors related to seeking treatment of fever for children under five by caregivers in the public and private health sector in Madagascar, Mali, and Nigeria.

### Conceptual model

**Fig 1:**
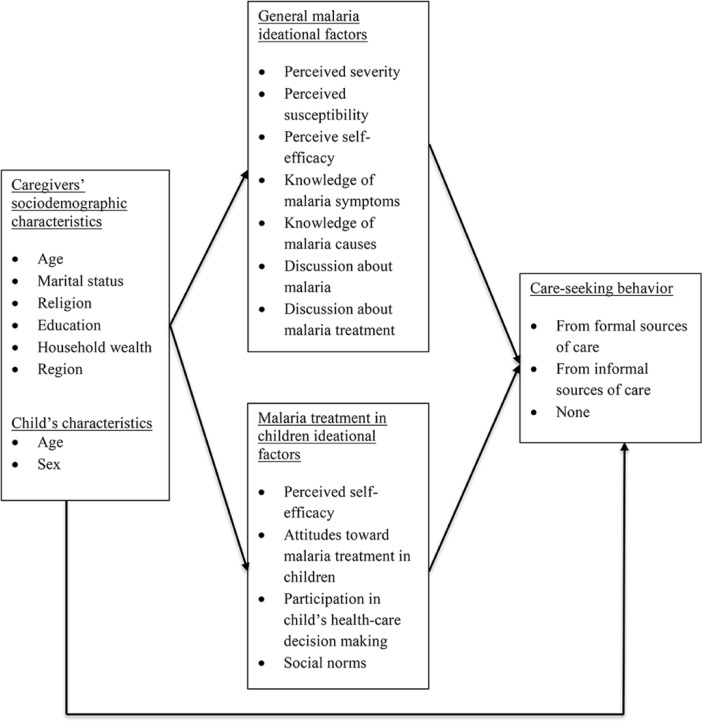
Malaria-related ideational factors and care-seeking behavior for fever among children under five.

This analysis is guided by Kincaid’s Ideation Model of Strategic Communication and Behavior Change [20], which posits that ideation is an intermediate construct, or behavioral determinant, between contextual factors and behavior. Ideation is comprised of three domains: cognitive, emotional, and elements of social interactions. Within each domain are related factors that influence behavior change. For example, the cognitive domain includes knowledge, attitudes, perceived risk, subjective norms, and self-image; the emotional domain includes emotional response, empathy, and self-efficacy; and the social interactions elements include social support, social influence, interpersonal communication, and personal advocacy [20–22]. We have adapted Kincaid’s model for this analysis. These factors are listed in Appendix 1 and described in detail below (Fig 1).

## Methods

### Sampling design

Data from Madagascar, Mali, and Nigeria were collected from household surveys in 2014 and 2015 under two projects funded by the U.S. Agency for International Development (USAID). Data from Madagascar and Nigeria were collected by the Health Communication Capacity Collaborative (HC3) project, while data from Mali were collected by the Keneya Jemu Kan project. Multi-stage sampling was employed in each country, where clusters were selected from geographic areas, households selected from clusters, and female caregivers interviewed within selected households. Eligible households in both Nigeria and Madagascar were those with at least one child under five years old. In Mali, additional households were eligible. However, for the purposes of this paper, analysis was limited to surveyed Malian households with children under five years old in order to compare data across the three countries.

In Madagascar, the survey was conducted between September and November 2014 (dry season/ early rainy season) and focused on the four malaria transmission zones. Districts were selected randomly from each zone: Ambohimahasoa and Miarinarivo in the Highland zone; Brickaville and Manakara in the Equatorial zone; Marovoay, Morombe, and Bekily in the Tropical zone; and Ambovombe in the Sub-desert. Clusters were selected in each of the transmission zones with their probability of selection being proportional to their population size. Twenty households were randomly selected from each cluster from among eligible households. Within each household, data collectors randomly chose a child under the age of five and interviewed the child’s female caretaker. The final survey sample included 2,368 adult female caregivers of children under five.

In Mali, the survey was conducted between July and September 2015 (rainy season) and focused on Koulikoro, Sikasso, and Mopti regions as well as three communes within the District of Bamako. Clusters were selected in each region or commune with their probability of selection being proportional to their population size. Twenty-five households were randomly selected from eligible households within each cluster. All eligible women in each household were selected for participation in the individual interview but only those with children under five were included in this paper’s analysis. The final sample included 3,542 adult female caregivers of children under five.

In Nigeria, the survey was also conducted between July and September 2015 (rainy season) in Akwa Ibom, Kebbi, and Nasarawa states. Within each state, 60 clusters were randomly selected. An average of 20 households were selected from eligible households in each cluster. Eligible households were home to at least one child under five and their adult female caregiver. Data collectors randomly chose a child under the age of five and interviewed his/her female caregiver. The final survey sample was 3,611 adult female caregivers of children under five.

For the purpose of this study, we limited the analysis to female respondents who were caregivers of a child under five living in the same household who had fever within the two weeks prior to the interview. This information was obtained from “yes” or “no” response to the question: “Have any children under the age of five years in this household been sick with fever in the past two weeks?” If more than one child under five had recently had fever, the respondent was asked about the child who was most recently sick with fever. Based on this criterion, the study was limited to 771 female caregivers in Madagascar, 396 in Mali, and 679 in Nigeria.

### Variables

The outcome of interest was a caregiver’s source of treatment for fever among children under five within the last two weeks, which came from the question: “From where did you seek treatment for this child’s fever?” Responses to this question were a list of formal and informal sources of care. In this study, the former included public and private facilities, such as hospitals, health centers, dispensary, clinics, dispensaries; the latter included pharmacies, traditional healers, chemists, and other sources. The outcome was a binary variable indicating whether treatment was sought at a formal facility as opposed to treatment sought at an informal facility or no treatment sought at all.

The main independent variables were grouped into two categories: general malaria ideational factors and ideational factors related to malaria treatment in children (see Appendix 1). Under general malaria ideation, perceived severity, susceptibility, and self-efficacy to prevent malaria—key components of cognitive and emotional domains—were measured using a respondent’s agreement to multiple items. Several items were reverse-coded so a higher score would indicate a higher perceived severity, susceptibility, and self-efficacy. For example, responses to the statement, “You don’t worry about malaria because it can be easily treated,” were reverse-coded because a disagreement would indicate a higher perception of severity compared to an agreement. Each index was then dichotomized at median to higher and lower levels of perception of severity, susceptibility, and self-efficacy. Malaria-related knowledge and discussion about malaria as well as malaria treatment were binary variables indicating whether a respondent gave a correct response in terms of knowledge or stated that they discussed these topics within the past year.

The second group of variables—ideational factors related to malaria treatment among children—included four variables. Perceived self-efficacy was measured by the degree of agreement with two statements relating to a caregiver’s perceived ability to recognize malaria among children without a health-care provider (see Appendix 1), except in Mali, where only the second statement was available. The second variable of this group, “attitudes toward malaria treatment among children,” is binary, indicating a respondent’s agreement to the statement: “[A] health worker is the best person to say if a child may have malaria.” Respondent involvement in health-care decision making for the child with fever was represented by a binary variable indicating whether the respondent made the decision by herself or jointly with her spouse. Finally, “social norms relating to malaria treatment in children” was represented by whether a caregiver believed that at least half of the children in her community visited a health-care provider on the same day a child developed a fever; this question was also not asked in Mali.

### Analysis

Separate analysis was conducted for each country. Within each country, descriptive and bivariate analyses were run to assess the outcome and its associations with the independent variables and key sociodemographic characteristics of caregiver respondents. Multivariate analysis was conducted to examine the associations of independent ideational variables with the outcome while controlling for respondent characteristics. We controlled for respondent exposure to media (television and radio), sociodemographic characteristics and, in Mali and Nigeria, the child’s age and gender (these variables were not available in the Madagascar dataset). Because of the small study samples, five household wealth quintiles were constructed for the entire samples and these five categories were then collapsed into three groups: the poorest and poor were combined to the first group, the middle group remained as the second group, and the rich and richest were combined into the third group. Because of the multi-stage sampling of the surveys, two-level models were run using the *melogit* command to take into account the sampling design—where households were nested within clusters. The empty models, which only included the cluster identification, showed some evidence of intraclass correlation, further justifying the use of the two-level models. All analyses were unweighted, as sampling weights were not available, and conducted using Stata version 13/SE [23].

### Ethical approval and informed consent

Ethical approval for each of the survey was obtained from the Johns Hopkins School of Public Institutional Review Board and the respective country authorities: the Madagascar Ministry of Public Health, the National Health Research Ethics Committee in Nigeria, and Mali Institutional Review Board. Interviewers obtained informed consent from all participants prior to the interviews; verbal consent was obtained in Madagascar and Nigeria, while, consistent with directives from the local IRB, written consent was obtained in Mali.

## Results

### Sample characteristics

In Mali, the sample was primarily aged 34 years or younger (82.4%) and almost everyone was married and Muslim (see Table 1). Two-thirds of the sample reported having no education, and a similar proportion lived in rural areas. The wealth distribution varied: the poor accounted for 38.5% of the sample, the middle group 14.9%, and the rich group 46.6%. The average age of the children under five being cared for was about one and a half years old and their sex was equally distributed between boys and girls.

**Table 1:** Sample distribution by caregiver’s characteristics.

	Mali	Nigeria	Madagascar
	(n = 396)	(n = 679)	(n = 572)
	% or mean (SD)	% or mean (SD)	% or mean (SD)
***Socio-demographic variables***			
Respondent’s age			
34 and younger	82.39	80.71	74.13
35 and older	17.61	19.29	25.87
Currently married			
No	3.28	5.89	20.98
Yes	96.72	94.11	79.02
Religion			
Christian and others	2.99	62.00	72.55
Muslim	97.01	38.00	27.45
Education (Mali’s categories in parentheses)			
No schooling	64.18	30.49	32.87
Primary (Fondamental 1)	17.91	30.34	45.45
Secondary (Fondamental 2)	12.24	34.90	16.96
Higher (Secondaire +)	5.67	4.27	4.72
Household wealth			
First	38.51	44.62	39.34
Second	14.93	---	20.80
Third	46.57	55.38	39.86
Age of index child (years)	1.42 (1.15)	2.34 (2.07)	---
Gender of index child			
Boy	48.96	50.52	---
Girl	51.04	49.48	---
Residence			
Rural	63.88	76.35	---
Urban	36.12	23.65	---
Region			
Koulikoro	32.84		
Sikasso	12.84		
Mopti	26.57		
Bamako	27.76		
State			
Akwa Ibom		39.03	
Kebbi		13.99	
Nasarawa		46.98	
District			
Miarinari			11.89
Ambohimah			6.99
Manakara			19.58
Brickaville			9.27
Marovoay			13.99
Morombe			9.09
Ambovombe			22.20
Bekily			6.99
***Exposure to the media***			
Watched TV at least once a week			
No	49.25	59.06	90.91
Yes	50.75	40.94	9.09
Listed to the radio at least once a week			
No	40.00	41.38	51.75
Yes	60.00	58.62	48.25

The sample of female caregivers in Nigeria was similar to those sampled in Mali, as they tended to be younger, and nearly everyone was married. Over three-fifths (62.0%) of the respondents were Christian and the remaining two-fifths (38.0%) were Muslim. Except for a few people with higher education, the caregivers in Nigeria were equally divided between having no education, primary schooling, and secondary schooling. More than half (55.4%) of respondents were considered in the rich household wealth category. The average age of the children under five being cared for was a little older than two years old, with equal numbers of boys and girls.

In Madagascar, the sociodemographic characteristics of the female caregiver sample was more mixed. Nearly one-third of the respondents were aged 35 years or older. About one-fifth (21.0%) of respondents were not married at the time of the survey, and Muslim respondents accounted for over a quarter of the sample. More than three-quarters (78.3%) of the sample had either no education or only primary schooling; very few went beyond secondary school. One-in-five respondents was in the middle wealth group, while the rest were equally divided between the rich and the poor groups.

Respondents in Mali reported the highest prevalence of media exposure among the three countries: about half (50.8%) of caregivers reported watching television at least once a week and two-fifths (60.0%) reported listening to the radio at least once a week. While Nigerian respondents reported lower exposure to television (40.9%), their exposure to the radio (58.6%) was similar to caregivers in Mali. In contrast, caregivers in Madagascar had the lowest prevalence of media exposure among the three countries: a mere 9.1% of caregivers reported watching television at least once a week and less than half reported listening to the radio.

### Ideational factors

#### General malaria ideational factors

In Mali, the prevalence of the various positive malaria ideational factors was fairly high. The vast majority (90.2%) of respondents gave the correct response about mosquito bites being the cause of malaria, and almost two-thirds (61.2%) were correct about fever being a malaria symptom (Table 2). Nearly two-thirds (63.9%) of the sample perceived a high level of severity of malaria, while 69.6% reported high perceived susceptibility to malaria. Perceived self-efficacy regarding the general ability to protect oneself against malaria was comparatively low as 58.5% of respondents reported a high level of self-efficacy.

**Table 2:** Ideational factors related to malaria and malaria treatment among children under five.

	Mali	Nigeria	Madagascar
	(n = 396)	(n = 679)	(n = 572)
	%	%	%
**General malaria ideational factors**			
Perceived severity			
Low	36.12	48.16	42.31
High	63.88	51.84	57.69
Perceived susceptibility			
Low	30.45	41.38	49.78
High	69.55	58.62	51.22
Perceived self-efficacy for protection against malaria			
Low	41.49	49.93	30.07
High	58.51	50.07	69.93
Knowledge of malaria symptom			
No	38.81	0.00	28.32
Yes	61.19	100.00	71.68
Knowledge about causes of malaria			
No	9.85	7.07	16.43
Yes	90.15	92.93	83.57
Discussion about malaria			
No	---	25.77	68.18
Yes	---	74.23	31.82
Discussion about malaria treatment			
No	---	63.48	83.74
Yes	---	36.52	16.26
Perceived self-efficacy in detecting malaria in children			
Low	33.73	33.73	29.37
High	66.27	66.27	70.63
Attitudes toward malaria treatment in children			
Low	5.07	41.24	4.90
High	94.93	58.76	95.10
Participation in health care decision making for child			
No	72.84	42.12	30.24
Yes	27.16	57.88	69.76
Social norms relating to malaria treatment in children			
Low	---	47.13	22.38
High	---	52.87	77.62

The samples in Nigeria and Madagascar showed greater heterogeneity in the prevalence of the various malaria ideational factors. For example, although knowledge of the cause of malaria and its symptoms was almost universal in Nigeria (e.g., all surveyed caregivers reported that mosquito bites were a cause of malaria and 92.9% knew that fever was a malaria symptom), only one-in-two Nigerian caregivers reported a high self-efficacy for malaria prevention. Just over half of the Nigerian sample reported a high level of perceived severity (51.8%) or perceived susceptibility (58.6%). In Madagascar, the majority of respondents correctly identified the cause of malaria (83.6%) and malaria symptoms (71.7%). More than half (57.7%) of women reported a high perceived severity of malaria, while almost three-quarters (69.9%) reported having high self-efficacy. Similarly, only one-in-two respondents in Madagascar believed that people were highly susceptible to malaria, which makes sense given that sample included caregivers from low malaria transmission zones.

#### Ideational factors related to malaria treatment in children

In Mali, about two-thirds (66.2%) of respondents reported that they could tell if a child had a typical or serious case of malaria, but nearly everyone (94.9%) agreed that a health-care worker was the best person to say whether a child had malaria. It was quite alarming that less than a quarter of the respondents had any say in making health-care decisions for their children.

In Nigeria, two out of three (66.3%) respondents reported having a high perceived self-efficacy in detecting malaria in children, and almost three-fifths (58.8%) reported that a health-care worker was the best person to make a diagnosis. A similar proportion (57.9%) reported participating in making health-care decisions for their children. Just over half (52.9%) believed that the majority of children in their community were brought to a health facility on the same day as the onset of fever.

In Madagascar, the ideational factors related to malaria treatment in children seemed slightly better, compared to the other two study countries. The prevalence of caregiver self-efficacy in detecting malaria in children and attitudes toward treatment were similar to findings in Mali. However, unlike Mali, nearly 70.0% of respondents in Madagascar reported having some say in making health-care decisions for their children. Over three-quarters (77.6%) of caregivers reported that bringing febrile children promptly to a health facility was a norm in their community.

### Associations between ideational factors and care-seeking

Among the three countries, Mali had the highest proportion (73.1%), followed by Nigeria (57.0%) and Madagascar (41.6%), of caregivers who sought care from a formal health-care provider for febrile children under five. The results of the multivariable regressions of general and malaria treatment-related ideational factors (adjusted odds ratios [ORs]) largely mirrored those of the bivariate analysis (unadjusted ORs), presented in Table 3.

**Table 3:** Ideational factors related to care-seeking from a formal health facility for children under five who had fever in the last two weeks.

	Mali		Nigeria		Madagascar	
	%	OR (s.e.)	%	OR (s.e.)	%	OR (s.e.)
Sought treatment from a formal health facility	73.13		57.00		41.61	
**General malaria ideational factors**						
Perceived severity						
Low	75.21	1.00	55.35	1.00	38.84	1.00
High	71.96	.69 (.22)	58.52	1.24 (.27)	43.64	1.29 (.29)
Perceived susceptibility						
Low	78.43	1.00	61.21	1.00	39.07	1.00
High	70.82	.65 (.22)	54.02*	.74 (.15)	44.03*	1.21 (.27)
Perceived self-efficacy for protection against malaria						
Low	71.22	1.00	52.80	1.00	36.05	1.00
High	74.49	1.19 (.37)	61.18	.90 (.20)	44.00	1.51 (.36)
Knowledge of malaria symptom						
No	73.08	1.00	(a)	(a)	42.59	1.00
Yes	73.17	.82 (.26)	(a)	(a)	41.22	.99 (.23)
Knowledge about causes of malaria						
No	45.45	1.00	45.83	1.00	34.04	1.00
Yes	76.16**	6.77 (3.37)**	57.84	1.07 (.41)	43.10	1.38 (.41)
Discussion about malaria						
No	---	---	54.86	1.00	42.56	1.00
Yes	---	---	57.74	1.38 (.35)	39.56	.71 (.18)
Discussion about malaria treatment						
No	---	---	57.31	1.00	41.96	1.00
Yes	---	---	56.45	1.15 (.26)	39.78	.95 (.30)
**Malaria treatment in children ideational factors**						
Perceived self-efficacy in detecting malaria in children						
Low	71.68	1.00	54.59	1.00	39.88	1.00
High	73.87	1.22 (.38)	58.22	1.23 (.27)	42.37	1.07 (.12)
Attitudes toward malaria treatment in children						
Low	70.59	1.00	53.57	1.00	39.29	1.00
High	73.27	1.09 (.73)	59.40	1.37 (.29)	41.73	.90 (.44)
Participation in health care decision making for child						
No	74.18	1.00	58.39	1.00	41.04	1.00
Yes	70.33	.74 (.24)	55.98***	.81 (.17)	41.85*	1.11 (.33)
Social norms relating to malaria treatment in children						
Low	---	---	48.75	1.00	32.81	1.00
High	---	---	54.35	2.51 (.52)***	44.14	1.76 (.45)*
# of clusters	104		161		117	
# of households	335		679		572	
ICC						
Cluster	.078		.18		.16	
AIC	400.6		819.77		787.03	

*p < .05

** p < .01

*** p < .001

--- data is not available.

(a) Variable excluded because there was not enough variability

Models controlled for socio-economic characteristics, exposure to the media, region (Mali), state (Nigeria), and district (Madagascar).

In Mali, controlling for a number of other ideational and sociodemographic factors, the odds of seeking care from the formal health sector for children who had fever within the last two weeks was more than six times higher among caregivers who knew what caused malaria compared to the odds of care-seeking among caregivers who did not know (p < .01). Social norms relating to malaria treatment of children were consistently important in Nigeria and Madagascar. Those female caregivers who believed that it was their community norm to promptly seek care for children with fever were much more likely to seek care from the formal health sector than those who did not (OR = 2.51, p < .001 in Nigeria; OR = 1.76, p < .05 in Madagascar).

Several sociodemographic characteristics were found to be significantly associated with seeking care for children with fever in the formal health-care sector, although the results were not consistent across countries. In Nigeria, respondents who were Muslim reported much lower odds of formal sector care-seeking, compared to similar caregivers of other religions (OR = .49; p < .05). Additionally, urban residents in Nigeria were twice more likely to seek care in the formal sector than rural residents (p < .05). In Madagascar, female caregivers who had attained the highest level of education (i.e., secondary and above) were the only group who reported significantly higher odds of seeking care in the formal sector for children with fever, compared to those with no education (OR = 2.65, p < .05).

## Discussion

This paper describes care-seeking behavior among adult female caregivers of children under five who had a fever within two weeks prior to the survey based on household surveys in three countries: Madagascar, Mali, and Nigeria. We examined whether the sick child was brought to a formal health facility for fever treatment and how this behavior might be related to the caregiver’s beliefs, attitudes, and perceived norms about malaria in general and, more specifically, about malaria treatment among children. We found a wide variation in the prevalence of care-seeking behaviors across study countries as well as variation in what ideational factors were significantly associated with care-seeking in the formal health sector. It should be noted that we conducted the same analyses among children under five who had fever in the last six months in Nigeria and Madagascar, where data were available, and found similar results.

Few ideational factors related to malaria and malaria treatment among children were significantly associated with care-seeking for fever among children under five in all three study countries. In Mali, knowing that malaria was caused by mosquito bites was associated with increased odds that a sick child was brought to a formal health facility for care. Knowledge of malaria symptoms was not associated with childhood malaria care-seeking in Mali and Madagascar. This finding contrasts with previous findings that suggested strong associations between malaria symptom knowledge and prompt and adequate care [13, 24–29], and suggests that program interventions that aim to increase malaria-related general knowledge alone may not be effective across countries. Such messages need to be tailored to each context in order to encourage caregivers to bring a sick child with fever to a formal health sector provider in these countries.

In addition, we did not find evidence of consistent associations between perceived severity, susceptibility, and self-efficacy related to malaria or malaria treatment among children with formal sector health-care-seeking. This is also contrary to previous studies [24, 30]. One should use caution when interpreting the results on perceived susceptibility, as they might vary depending on the time of year the survey took place. For example, during the dry season, people may feel less at risk for being bitten by a mosquito and, therefore, may report their perceived susceptibility as lower than they might report if they completed a questionnaire during the rainy season. This is true in Madagascar, where the survey was partially conducted in the dry season and we found the lowest proportion of survey respondents reporting high perceived susceptibility compared to the other two countries. It may also explain the highest proportion of respondents reporting perceived self-efficacy in malaria protection but the lowest proportion of those reporting knowledge of malaria causes and discussion about malaria and malaria treatment in Madagascar, compared to Mali and Nigeria. Consequently, there may be less variation in perceived susceptibility across transmission zones and associations between perceived susceptibility and care-seeking are less likely to be found. In Nigeria and Madagascar, the belief that the majority of children with malaria were brought for care promptly was significantly related to care-seeking at a formal sector facility. This measure, unfortunately, was not collected in Mali. Had it been collected in the Mali survey, we anticipate that it may also have a positive association with care-seeking, although the magnitude of the associations might not have been as strong as it was in Nigeria and Madagascar, because care-seeking behavior was more prevalent in Mali (73.1%) than in the other two study countries (57% and 41.6%, respectively). This finding underscores the importance of social influence on care-seeking behavior, relative to the influence of one’s own knowledge and perceptions related to the health condition. This finding suggests that communication efforts that use modeling to convey the message that prompt care-seeking for fever is the norm in the community are likely to be effective in promoting appropriate care-seeking for fever.

It is interesting to note that a female caregiver’s involvement in making decisions about the health care of the child was not related to the outcome in any of the countries in this study, despite findings from several previous studies that highlighted gender dynamics within households as an important factor influencing whether a sick child would be brought for care [13, 31, 32]. It is possible that our measure of decision making is an overly simplified measure of gender dynamics within households. All caregivers in the current analysis were women who, Ellis et al. [31] suggested, were often the first one to identify illness symptoms. However, decisions about care-seeking were often made by fathers and other senior members of the household and can vary greatly between households [30, 33]. In other words, an empowered woman might still face serious barriers preventing her from acting promptly when the health of a child is at stake. Franckel and Lalou [32] also observed this phenomenon in rural Senegal and suggested that family management of childhood malaria is often aimed at making the best use of household financial and resources, as well as of the availability and functionality of the mother, father, and other relatives in the household. Such household dynamics could result in favoring home-based care and delaying seeking care at health facilities [30, 32]. If this is true in our study countries, it suggests that early involvement of fathers in child health care should be emphasized in future programs. Program efforts should also include components designed not only to promote the power of female caregivers in decisions about child health care but also to educate and engage men to seek care promptly when young children are sick with fever. Unfortunately, the number of male caregivers in the surveys was too small and quantitative data collected were limited to allow further examinations of this topic.

Several factors that may influence care-seeking were not examined in this study. First, in addition to social norms mentioned above, measures of malaria-related discussion were not collected in Mali, thus it is not possible to assess the associations between them and care-seeking in this country. Second, other studies have indicated a strong link between physical access—distance or travel time to a provider—and care-seeking for malaria [13, 34–39]. It is possible that seeking care for children under five with fever in these three countries depends more on supply-side than on demand-side factors. Unfortunately, our surveys did not collect information on the health service environment at the community level, which can be a direction for future research. For example, Bedford and Sharkey [13] reported how study participants in Nigeria described limited geographic access, frequent stock-outs of medicines, the unclean environment of health facilities, and negative attitudes of health providers as important barriers to care-seeking for childhood pneumonia, diarrhea, and malaria. Similar results have also been reported elsewhere in sub-Saharan Africa [40, 41]. Other limitations of the study include the cross-sectional nature of the data, which prohibits the inference of a cause-effect relationship between caregiver ideation and their care-seeking behavior. It is also possible that respondent responses were affected by social desirability bias.

Despite the limitations, the study highlights a wide variation in caregiver care-seeking practices for children under five with fever across the three study countries. Care-seeking from a formal health-care provider is far from a norm for children under five with fever in these countries. Although knowledge is generally high, there is still some evidence of knowledge being essential to malaria care-seeking, yet intervention messages need to be tailored to the specific country in order to be effective. Program interventions that aim to promote care-seeking through targeting social influences are also likely to result in increased care-seeking in the formal sector for children under five with fever. Finally, it may be worthwhile for future research to explore gender and family dynamics in decision making about child health and their role in care-seeking behavior for children under five with fever.

## Supporting information

S1 File List of ideation variables. (DOCX)

## Abbreviations

ACTs: artemisinin-based combination therapies
DHS: demographic and Health Survey
PMVs: patent medicine vendors
PMI: President’s Malaria Initiative
WHO: World Health Organization
