# Supplementary material for: Associations between malaria-related ideational factors and care-seeking behavior for fever among children under five in Mali, Nigeria, and Madagascar
Source: PLoS One. 2018 Jan 25;13(1):e0191079. doi: 10.1371/journal.pone.0191079 (PMC5784922; doi:10.1371/journal.pone.0191079)
Supplement: S1 File — (DOCX) [file pone.0191079.s001.docx]

**Appendix 1. List of ideation variables**

1. **General malaria ideational factors**
2. Perceived severity: the degree of agreement to the following statements
   1. You don’t worry about malaria because it can be easily treated
   2. Every case of malaria can potentially lead to death
   3. When someone you know gets malaria, you usually expect them to completely recover in a few days
   4. When your child has fever, you usually wait a couple of days before going to a health provider
   5. Only weak children can die from malaria
3. Perceived susceptibility: the degree of agreement to the following statements
   1. During the rainy season, you worry almost every day that someone in your family will get malaria
   2. When your child has a fever, you almost always worry that it might be malaria
   3. People in this community only get malaria during rainy season
   4. People only get malaria when there are lots of mosquitos
   5. You cannot remember the last time someone you know became dangerously sick with malaria
4. Perceived self-efficacy for protection against malaria: the degree of certainty in the followings
   1. Easily protect yourself from getting malaria
   2. Easily protect your children from getting malaria
5. Knowledge of malaria symptom: respondent knew that fever is a symptom of malaria
6. Knowledge about causes of malaria: respondent knew that mosquito bites are causes of malaria
7. Discussion about malaria: respondent discussed malaria with friends and relatives (except spouse) in the past year
8. Discussion about malaria treatment: respondent discussed malaria treatment with spouse, friends, or relatives (anyone) in the past year
9. **Malaria treatment in children ideational factors**
10. Perceived self-efficacy in detecting malaria in children: the degree of agreement or certainty to the followings:
    1. Sometimes, parents will know that a child has malaria even if a health provider’s test says that he or she does not have malaria (not available in Mali)
    2. Know if a child has a typical or serious case of malaria
11. Attitudes toward malaria treatment in children: the degree of agreement to the following
    1. Health worker is the best person to say if a child may have malaria
12. Participation in health-care decision making for child: respondent made decision alone or jointly with spouse
13. Social norms relating to malaria treatment in children (not available in Mali): respondent’s perception that at least half of the children in the community visit a health provider on the same day that they develop a fever
